# Intra-genome variability in the dinucleotide composition of SARS-CoV-2

**DOI:** 10.1093/ve/veaa057

**Published:** 2020-08-13

**Authors:** Paul Digard, Hui Min Lee, Colin Sharp, Finn Grey, Eleanor Gaunt

**Affiliations:** Department of Infection and Immunity, The Roslin Institute, The University of Edinburgh, Easter Bush Campus, Midlothian, EH25 9RG, UK; Department of Infection and Immunity, The Roslin Institute, The University of Edinburgh, Easter Bush Campus, Midlothian, EH25 9RG, UK; Department of Infection and Immunity, The Roslin Institute, The University of Edinburgh, Easter Bush Campus, Midlothian, EH25 9RG, UK; Department of Infection and Immunity, The Roslin Institute, The University of Edinburgh, Easter Bush Campus, Midlothian, EH25 9RG, UK; Department of Infection and Immunity, The Roslin Institute, The University of Edinburgh, Easter Bush Campus, Midlothian, EH25 9RG, UK

**Keywords:** SARS-CoV-2, CpG dinucleotides, virus evolution

## Abstract

CpG dinucleotides are under-represented in the genomes of single-stranded RNA viruses, and SARS-CoV-2 is no exception to this. Artificial modification of CpG frequency is a valid approach for live attenuated vaccine development; if this is to be applied to SARS-CoV-2, we must first understand the role CpG motifs play in regulating SARS-CoV-2 replication. Accordingly, the CpG composition of the SARS-CoV-2 genome was characterised. CpG suppression among coronaviruses does not differ between virus genera but does vary with host species and primary replication site (a proxy for tissue tropism), supporting the hypothesis that viral CpG content may influence cross-species transmission. Although SARS-CoV-2 exhibits overall strong CpG suppression, this varies considerably across the genome, and the Envelope (E) open reading frame (ORF) and ORF10 demonstrate an absence of CpG suppression. Across the *Coronaviridae*, E genes display remarkably high variation in CpG composition, with those of SARS and SARS-CoV-2 having much higher CpG content than other coronaviruses isolated from humans. This is an ancestrally derived trait reflecting their bat origins. Conservation of CpG motifs in these regions suggests that they have a functionality which over-rides the need to suppress CpG; an observation relevant to future strategies towards a rationally attenuated SARS-CoV-2 vaccine.

## 1. Introduction

CpG dinucleotides are under-represented in the DNA genomes of vertebrates (Cooper and Krawczak 1989; Simmonds et al. 2013). Cytosines in the CpG conformation may become methylated, and this methylation is used as a mechanism for transcriptional regulation (Medvedeva et al. 2014). Methylated cytosines have a propensity to undergo spontaneous deamination (and so conversion to a thymine). Over evolutionary time, this has reduced the frequency of CpGs in vertebrate genomes (Cooper and Krawczak 1989). However, loss of CpGs in promoter regions would affect transcriptional regulation, and so CpGs are locally retained, resulting in functionally important ‘CpG islands’ found in around half of all vertebrate promoter regions (Deaton and Bird 2011).

Single-stranded RNA (ssRNA) viruses infecting vertebrate hosts reflect the CpG dinucleotide composition of their host in a type of mimicry (Simmonds et al. 2013). It was hypothesised that this is because vertebrates have evolved a CpG sensor which flags transcripts with aberrant CpG frequencies (Atkinson et al. 2014; Gaunt et al. 2016). This idea was strengthened by the discovery that the cellular protein Zinc-finger Antiviral Protein (ZAP) binds CpG motifs on viral RNA and directs them for degradation (Takata et al. 2017) and further supported by observations that CpGs can be synonymously introduced into a viral genome to the detriment of virus replication without negatively impacting transcriptional or translational efficiency (Tulloch et al. 2014; Gaunt et al. 2016). Current understanding is therefore that ssRNA viruses mimic the CpG composition of their host at least in part to subvert detection by ZAP. ssRNA viruses also under-represent the UpA dinucleotide, but to a far more modest extent (Simmonds et al. 2013), and the reasons behind UpA suppression are less well understood. A consequence of dinucleotide bias is that certain codon pairs are under-represented (Tulloch et al. 2014; Kunec and Osterrieder 2016) (so, e.g. codon pairs of the conformation NNC-GNN are among the most rarely seen codon pairs in vertebrates; Tats et al. 2008)). Whether the two phenomena of CpG suppression and codon pair bias (CPB) are discrete remains controversial (Futcher et al. 2015; Groenke et al. 2020; Kunec and Osterrieder 2016).

The *Coronaviridae* have a generally low genomic cytosine content (Berkhout and van Hemert 2015), but as with other ssRNA viruses, nonetheless still under-represent CpG dinucleotides to a frequency below that predicted from individual base frequencies of cytosine and guanine (Woo et al. 2007).

The Coronavirus family comprises four genera—the alpha, beta, gamma, and delta-coronaviruses. Human-infecting coronaviruses (HCoVs) have been identified belonging to the alpha and beta genera (Hu et al. 2015). Alphacoronaviruses infecting humans include HCoV-229E and the more recently discovered HCoV-NL63 (van der Hoek et al. 2004). Betacoronaviruses include HCoV-OC43, HCoV-HKU1 (Woo et al. 2005), severe acute respiratory syndrome (SARS)-CoV (Rota et al. 2003), Middle East respiratory syndrome (MERS)-CoV (Zaki et al. 2012) and the recently emerged SARS-CoV-2 (Lu et al. 2020; Zhu et al. 2020). Prior to the emergence of SARS-CoV-2, SARS-CoV had the strongest CpG suppression across HCoVs (Woo et al. 2007). The reason(s) for this are uncertain, but loss of CpG from a virus genome upon zoonotic transfer into the human host has previously been reported for influenza A virus (Greenbaum et al. 2008), potentially indicating an advantage of reduced CpG content for infection of the human respiratory tract. All HCoVs are thought to be derived from ancestral bat viruses, though intermediate hosts may have facilitated zoonotic passage in some cases (Banerjee et al. 2019).

During replication, coronaviruses synthesise transcriptionally active negative sense sub-genomic RNAs which are of varying length. Sub-genomic RNAs are synthesised by the viral polymerase copying the genome up to a 5′ leader sequence (Liao and Lai 1994) which is repeated upstream of most open reading frames (ORFs) in the coronavirus genome (such repeats are referred to as transcription regulation sequences (TRSs)); this complementarity allows viral polymerase jumping from the 5′ leader sequence to directly upstream of ORFs preceded by a TRS (Sawicki and Sawicki 1998). The negative sense sub-genomic RNAs serve as efficient templates for production of mRNAs (Sawicki et al. 2007). Generally, only the first ORF of a sub-genomic mRNA is translated (Perlman and Netland 2009), although leaky ribosomal scanning has been reported as a means for accessing alternative ORFs for several coronaviruses including SARS-CoV (Schaecher et al. 2007).

SARS-CoV-2 was recently reported to have a CpG composition lower than other members of the betacoronavirus genus, comparable to certain canine alphacoronaviruses; an observation used to draw inferences over its origin and/or epizootic potential (Xia 2020). Here, we show that coronaviruses have a broad range of CpG composition which is partially host and tissue tropism dependent, and that there is no difference in CpG content across coronavirus genera. There is however a striking disparity in CpG composition between SARS-CoV-2 ORFs, with the Envelope (E) protein ORF and ORF10 over-representing CpG dramatically. E ORF and ORF10 also have higher UpA dinucleotide composition and lower CPB scores than other ORFs. E ORF displays CpG suppression in all human-infecting viruses except SARS-CoV and SARS-CoV-2, suggesting a potential correlation between CpG presentation and disease severity in HCoVs.

## 2. Materials And Methods 

### 2.1 Sequences

**Figure 1.**
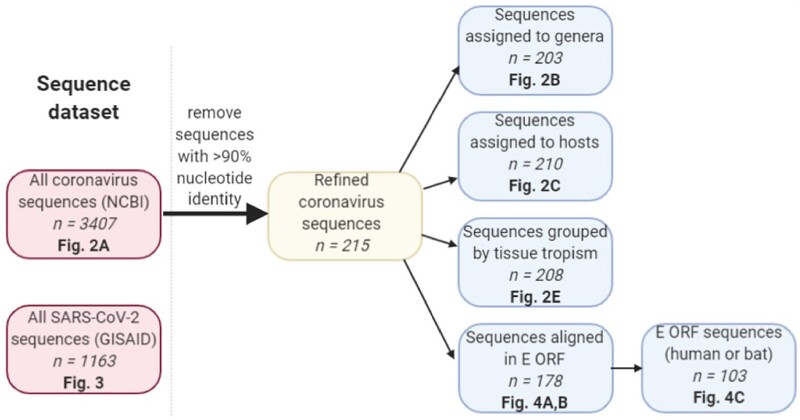
Workflow for sequence processing. Two sequence datasets were used for analysis; all coronavirus complete genome sequences available on NCBI, and SARS-CoV-2 complete genome sequences available on the GISAID platform (left-hand pink shaded boxes). The coronavirus complete genome sequences were cleaned by removal of sequences with 90 per cent nucleotide identity or greater to remove epidemiologic biases, leaving 215 complete genome sequences (central yellow shaded box). These were then categorised by genera, host and tissue tropism. The subset of 215 sequences were also aligned over the E ORF and grouped by host (blue shaded boxes). Each box first describes each dataset used, the number of sequences in that dataset is then indicated in *italicized* font, and the figure to which the dataset corresponds is indicated in bold font.

For a comparison of GC content versus CpG ratio, all SARS-CoV-2 complete genome sequences of high coverage (as defined on the GISAID website) were downloaded from GISAID (www.gisaid.org) on 26 March 2020 (1,163 sequences in total) and aligned against the SARS-COV-2 reference sequence (accession number NC_045512) using Simmonics software (Simmonds 2012) SSE v1.4 (pre-release download kindly provided by Prof. Peter Simmonds, Oxford University). All sequences represented human isolates except for one sequence of bat origin (hCoV-19/bat/Yunnan/RaTG13/2013; EPI_ISL_402131) and one sequence from a pangolin (hCoV-19/pangolin/Guangdong/1/2019; EPI_ISL_410721). All complete genome sequences of all coronaviruses were downloaded from NCBI on the 16 April 2020 (3,407 sequences in total). Sequences were then aligned and sequences <10 per cent divergent at the nucleotide level, identified using the ‘identify similar/identical sequences’ function in SSE v1.4 were removed from the dataset. Sequences were annotated into animal groups and genera based on their description in the NCBI database. The trimmed dataset (Supplementary Table S1) included 215 complete genome coronavirus sequences. Individual groups were made for sequences originating from the following hosts: bat (*n *=* *109), avian (35), camelid (3), canine (7), feline (9), human (7), mustelids (5), rodents (8), swine (13), ungulates (10) and ‘other’ (which included bottle-nosed dolphin (2), hedgehog (2), rabbit (2), beluga whale (1), civet (1) and pangolin (1)). Groups were loosely defined based on taxonomic orders, with some exceptions made to examine our specific research questions. Bats are of the order Chiroptera; multiple avian orders were grouped together (Galliformes, Anseriformes, Passeriformes, Gruiformes, Columbiformes and Pelicaniformes); even toed (Artiodactyla) and odd toed (Perissodactyla) ungulate orders were grouped, with camelids analysed separately due to their association with MERS-CoV (Azhar et al. 2014); Canidae (canine) and Pantherinae (feline) sequences of the Carnivora order were analysed separately, as canines have previously been suggested as an intermediate host species for SARS-CoV-2 (Xia 2020) and cat infections with SARS-CoV-2 have been reported (Shi et al. 2020); humans were the only representatives from the Primate order; all remaining Carnivora, with the exception of a single civet sequence, belonged to the Mustelidae (mustelids); rodents belong to the Rodentia order; and swine belong to the Artiodactyla order; whales are also Artodactyla but swine were considered separately due to considerable interest in porcine coronaviruses (Vlasova et al. 2020). Sequences were also annotated for genus by reference to the NCBI description (203 of the 215 sequences were assigned to a genus), and for primary replication site by literature reference (refer to Supplementary Table S1). Replication site annotations were based on the sample type from which a coronavirus sequence was obtained—‘enteric’ for faecal/gastrointestinal samples, ‘respiratory’ for nasal, oropharyngeal and other respiratory samples; ‘multiple’ if samples from multiple systems tested positive, ‘other’ if the sample was collected from a site not falling into the enteric or respiratory categories (e.g. brain), or ‘unknown’ if a sample type could not be determined. If only one sampling route was tested and returned a positive result, the sequence was categorised in accordance with the sole sampling route. The sequence datasets used in this paper is summarised in Fig. 1.

### 2.2 Analyses of dinucleotide content

CpG and UpA composition of complete genomes or of individual ORFs were calculated using the composition scan in SSE v1.4. CpG frequencies were measured as observed:expected (O:E) ratios, using the formula f(CpG)/f(C)*f(G). Individual ORFs were identified using a combination of ORF finder (https://www.ncbi.nlm.nih.gov/orffinder/), visual inspection of nucleotide alignments in SSE v1.4, comparison with previous literature and information available from nextstrain.org. Sliding window analyses were performed on the 1,163 aligned SARS-CoV-2 sequences and the related bat and pangolin sequences by performing composition scans in SSE v1.4 for 100 nucleotide genomic regions, at 25 nucleotide iterations. For the SARS-CoV-2 sequences, mean CpG O:E ratios for each window were calculated. CPB (Gutman and Hatfield, 1989) scores across the SARS-CoV-2 ORFeome were calculated using the SSE v1.4 composition scan function. Individual ORFs were concatenated with a separating ‘NNN’ codon for analysis, and secondary overlapping ORFs were not included due to coding constraints imposed in these regions.

To examine the extent of CpG retention in E ORF, the same analyses were performed with an additional correction for amino acid composition (Corr_CpG dataset produced by SSE v1.4).

### 2.3 Codon usage analysis

To examine the use of rare codons, codon adaptation index (CAI) values were calculated (https://www.biologicscorp.com/tools/CAICalculator).

### 2.4 Phylogenetic analyses

Of the 215 divergent sequences included in the analysis, E ORF could be identified in 178 by homology with E ORFs previously annotated in NCBI. Of these 178 E ORFs, seven were sequences isolated from humans and ninety-six were from bats; these sequences were selected for analysis. E ORFs were aligned in MEGA X (Kumar et al. 2018) using the Clustal method. Phylogenetic reconstruction was performed using an unrooted maximum likelihood tree, with gamma distributed variation in rates between branches and 100 bootstraps (also in MEGA X).

## 3. Results

### 3.1 CpG suppression within coronavirus genomes varies between host species and tissue tropism but not between genera

**Figure 2.**
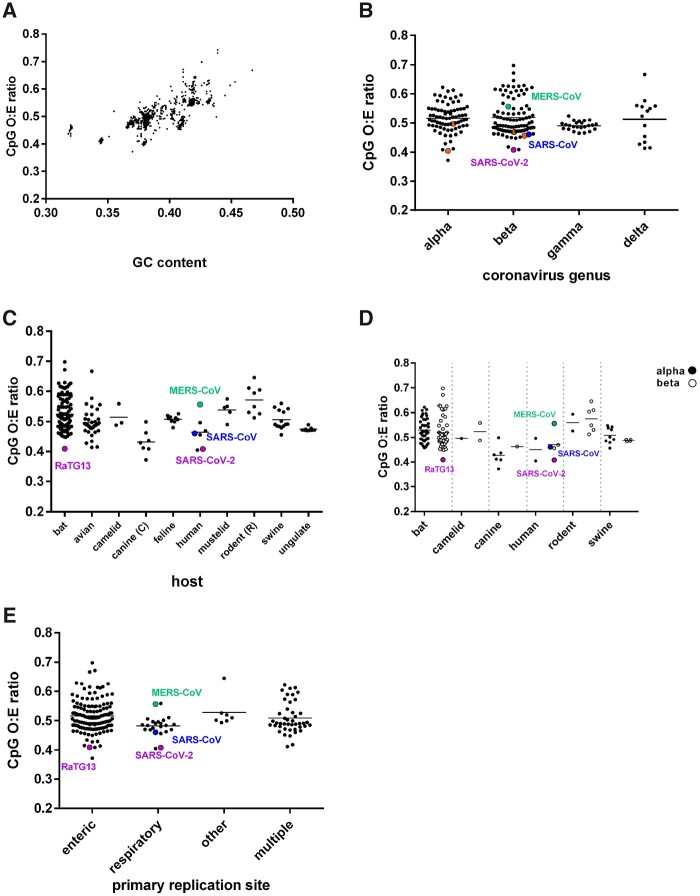
Comparison of the CpG ratios of complete genomes of coronaviruses. SARS-CoV is represented by a blue circle, SARS-CoV-2 and its related bat sequence RaTG13 by purple circles and MERS-CoV by a green circle throughout. A. GC content versus CpG ratio for all complete genome sequences of coronaviruses downloaded from Genbank (3407 sequences). The sequence dataset in (A) was then stripped to include only one representative from sequences with less than 10 per cent nucleotide diversity to overcome epidemiologic biases (215 representative sequences), which were analysed in the subsequent sub-figures. (B) Coronavirus genus against genomic CpG content. Other human-infecting coronaviruses HCoV-2292E, HCoV-NL63 (alphacoronaviruses) and HCoV-HKU1 and HCoV-OC43 (betacoronaviruses) are represented using orange circles. C. Vertebrate host of coronavirus against genomic CpG content. (D) Vertebrate host of coronavirus, with further sub-division into coronavirus genus, against genomic CpG content. Alphacoronaviruses are denoted with filled circles and betacoronaviruses with open circles. (E) Primary replication site against genomic CpG content by host. For a full breakdown of how these were assigned, please refer to Supplementary Table S1.

The genomic CpG composition of all complete genome coronavirus sequences (*n *=* *3,407; downloaded and further processed as described in the methods section and Fig. 1) were calculated using O:E ratios, with any value below 1 indicating CpGs are under-represented relative to the genomic content of cytosine and guanine bases. A substantial range in GC content (from ∼0.32 to 0.47) was seen across the *Coronaviridae*, and as expected, all viruses exhibited some degree of CpG suppression, with CpG O:E ratios ranging from 0.37 to 0.74 (Fig. 2A). To investigate the root of this variation, the coronavirus sequence dataset was refined to remove sequences with more than 90 per cent nucleotide identity to reduce sampling biases (so, e.g. SARS-CoV sequences of human origin were stripped from over 1,000 representative sequences to just one). The CpG compositions of the remaining 215 sequences (Supplementary Table S1) were compared between coronavirus genera (alpha, beta, gamma and delta). For the 215 representative sequences, a genus could be assigned for 203. No differences in CpG composition between coronavirus genera were apparent, although the gamma genus exhibited a tighter range (Fig. 2B). Next, we examined whether differences in CpG composition between viruses isolated from different hosts explained the range in CpG composition across the *Coronaviridae*. For the 215 representative sequences, a host could be assigned to 210. Coronavirus sequences were divided into host groups, and groups with at least three divergent sequences were compared; this included bat, avian, camelid, canine, feline, human, mustelid, rodent, swine and ungulate viruses. Variation in CpG composition between coronaviruses detected in different host species was evident across and between groups, with coronaviruses detected in canine and human species having lower CpG content and rodent and bat coronaviruses having the highest (Fig. 2C). All frequency ranges overlapped, however, indicating viral CpG frequency alone seems to be a poor predictor of virus origin, contradicting the recent suggestion of a canine origin of SARS-CoV-2 (Xia 2020). Where sequences in a host group representative of both alpha and betacoronaviruses were available (which was the case for bat, camelid, canine, human, rodent and swine viruses), these sequences were split by genus and compared to determine whether coronavirus genera influenced coronavirus CpG frequencies in a host species-specific manner. By this method, the lack of difference in CpG composition of coronaviruses of different genera was maintained (Fig. 2D).

To test the hypothesis that coronavirus CpG content varies according to tissue tropism (Xia 2020), we classified the viruses according to their primary site of replication, where this was known or could be inferred from the sampling route. Samples were split into five categories—‘respiratory’, ‘enteric’, ‘multiple’, ‘other’ or ‘unknown’. Altogether, 206 of the 215 sequences were classifiable (detailed in Supplementary Table S1), with nine sequences categorised as ‘unknown’ and excluded from further analyses. By this admittedly inexact approach, viruses infecting the respiratory tract had a lower mean CpG composition than viruses with enteric tropism (Fig. 2E). However, the spread of respiratory virus CpG frequencies was contained entirely within the range exhibited by enteric viruses. Furthermore, 124 sequences were assigned to the enteric group, and only twenty-two to the respiratory group. Of these 146 sequences, bat viruses accounted for 80, all of which were assigned to the enteric group (despite reasonable sampling of respiratory tract in bats) and this cohort of viruses maintained almost the full spread of CpG frequencies (Fig. 2E, Supplementary Table S1). Thus, while coronavirus CpG frequency may show some correlation with replication site, the dataset available does not permit strong conclusions to be drawn or predictions about zoonotic potential to be made.

### 3.2 Heterogeneities in the dinucleotide composition of SARS-CoV-2

**Figure 3.**
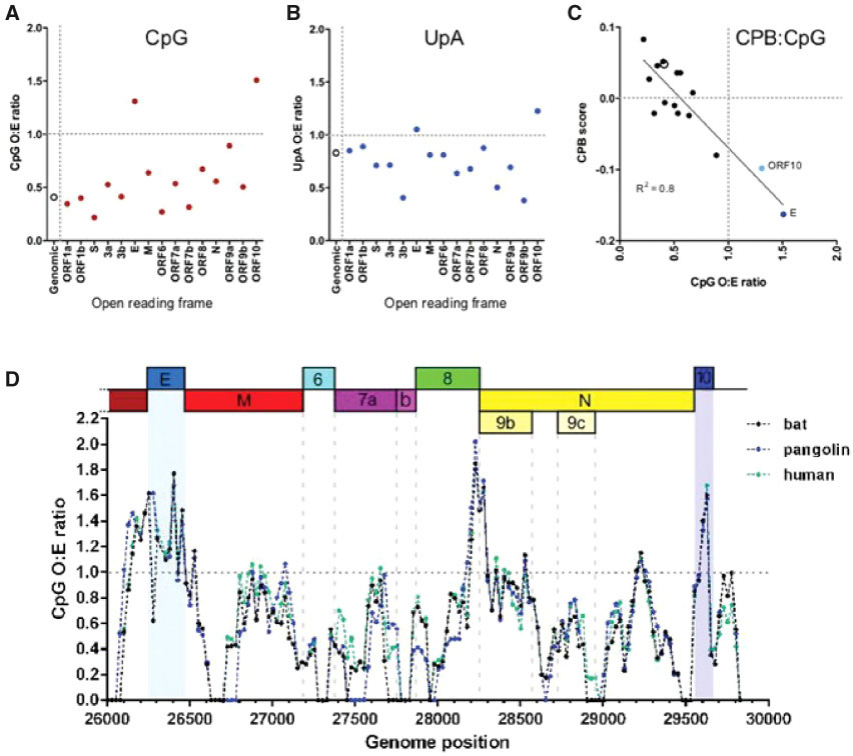
Heterogeneities in the dinucleotide composition of the SARS-CoV-2 genome. (A–C) Comparison of the dinucleotide and coding compositions of SARS-CoV-2 open reading frames (ORFs) for (A) CpG observed:expected (O:E) ratios, (B) UpA O:E ratios and (C) Codon pair bias (CPB) scores. Average scores across the genome are indicated using open circles. (D), Sliding window analysis of CpG content of SARS-CoV-2 (green line) and closely related bat (RaTG13; black line) and pangolin (purple line) isolates. The CpG O:E ratio of the 3′ end of the genome was measured in 100 nucleotide windows in 25 nucleotide increments. The mean of 1,163 complete genome sequences is presented for SARS-CoV-2.

By our methods for calculating CpG O:E ratios, SARS-CoV-2 has a genomic CpG ratio of 0.408 (representing the mean of 1,163 complete genome sequences). This is similar to the value calculated previously for a much smaller sample (*n *=* *5) of SARS-CoV-2 sequences (Xia 2020). As this previous study noted, this is at the bottom end of the range of genomic CpG O:E ratios for betacoronaviruses and for coronaviruses detected in humans (Figs 2B–D). However, as noted above, vertebrate DNA genomes contain localised islands of higher CpG content (Deaton and Bird, 2011). To determine if similar heterogeneity in CpG frequency was evident in the SARS-CoV-2 genome, the composition of individual ORFs was examined. Overall, most ORFs had CpG O:E ratios which were comparable to the genomic CpG ratio. However, two ORFs in particular, E ORF and ORF10, had CpG ratios higher than 1, indicating an absence of CpG suppression in those regions (Fig. 3A). These two ORFs also did not suppress the UpA dinucleotide, in contrast with other SARS-CoV-2 ORFs (Fig. 3B).

Due to the difficulties in distinguishing between dinucleotide bias and CPB, CPB scores were also calculated for each ORF and plotted against CpG composition (Fig. 3C). CPB scores provide an indication of whether the codon pairs encoded in each ORF are congruous with usage in vertebrate genomes. A score below 0 indicates use of codon pairs that are disfavoured in host ORFs. An approximately linear relationship between CpG O:E ratio and CPB score for each SARS-CoV-2 ORF was apparent (*R*^2^ = 0.80). E ORF and ORF10 both had negative CPB scores, indicating that they use under-represented codon pairs and in keeping with the observation that both ORFs over-represent CpG and UpA dinucleotides.

To examine the precise location of the CpG hotspots, a sliding window analysis of CpG content across the 3’ end of the SARS-CoV-2 genome (averaged over 1,163 complete genome sequences) as well as the closely related bat and pangolin sequences was performed. As expected, marked increases in CpG O:E ratio were observed concomitant with the genomic regions associated with E ORF and ORF10 (Fig. 3D). The E ORF and ORF10 regions associated with high CpG composition were maintained across the bat, pangolin and human sequences, indicating that since the bat sample was collected in 2013, the higher CpG frequency in this region has not been negatively selected. While the increase in CpG presentation was apparent across the entire E ORF, starting at the 3′ end of ORF3 and ending at the beginning of the M gene, the CpG spike in ORF10 was more narrowly associated with the putative coding region. Additionally, a CpG spike between the 3′-end of ORF8 and the 5′-end of the N gene was evident. The 5′-end of the N ORF also contains the overlapping ORF9b gene, which when considered alone, has a CpG O:E ratio approaching 1 (Fig. 3A), and is the ORF with the third-highest CpG O:E ratio after E ORF and ORF10. The usual coding plasticity afforded to nucleotides in the third position of a codon is nullified when overlapping reading frames are present, and so the CpG spike at this gene boundary is not surprising. Thus, although the SARS-CoV-2 genome exhibits high CpG suppression overall, there are local heterogeneities associated with individual ORFs, most notably E.

### 3.3 On the origins of the high CpG content of E ORF of SARS-CoV-2

**Figure 4.**
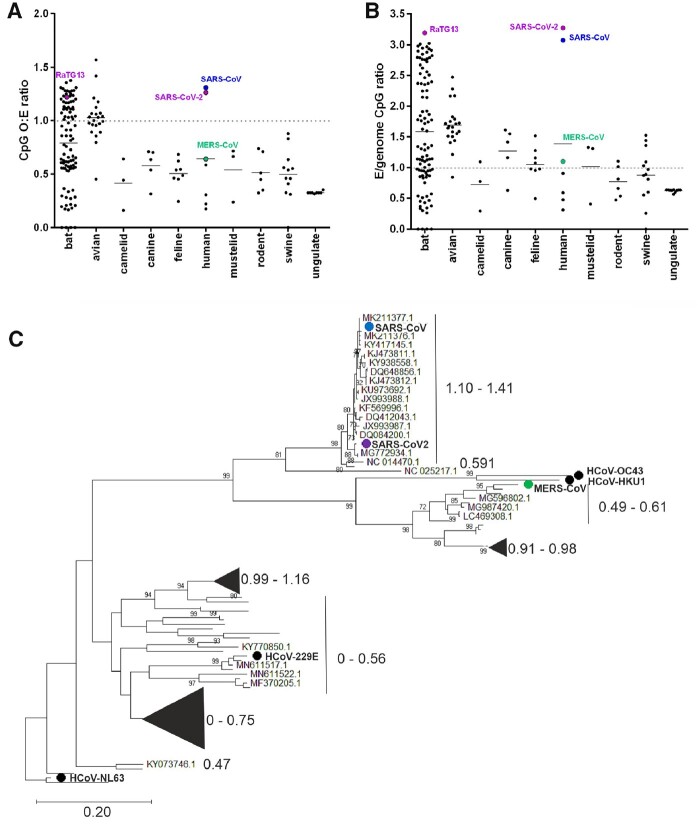
Evolutionary conservation of E ORF CpG content. MERS-CoV (green circle), SARS-CoV (blue circle) and SARS-CoV-2 and its bat sequence relative RaTG13 (purple circles) are indicated in all panels. (A), CpG O:E ratios for E ORF for 178 coronavirus E ORFs are plotted by host. (B), CpG O:E ratios for E ORF were divided by the genomic CpG O:E ratio for 178 coronavirus sequences and grouped by host. (C), Phylogenetic reconstruction of E ORF of human and bat coronaviruses. Maximum composite likelihood tree (100 bootstraps) representing the seven human-infecting coronaviruses (HCoV-229E, HCoV-HKU1, HCoV-NL63, HCoV-OC43 are indicated by black circles) and 96 bat coronaviruses for which E ORF could be identified by alignment with the human coronaviruses. CpG O:E ratios for the E gene are indicated by large font numbers, and the sequences to which they relate are either bracketed or represented by triangles scaled to indicate the number of sequences they represent.

To determine whether the high CpG content of E ORF is evolutionarily conserved (ORF10 is poorly conserved and only encoded by a subset of SARS-like coronaviruses, so it was not analysed), attempts to identify the E ORF by nucleotide alignment for the set of 215 coronavirus sequences was undertaken, compared with E ORFs already annotated in NCBI. Of the 215 sequences, E ORF was identifiable in 178, with the remaining sequences too divergent to be confident of gene assignment. CpG composition for E ORF for the 178 sequences was measured and plotted according to host. Amino acid conservation within the short ORF of E could bias levels of CpG; for example, amino acids encoded by codons containing C and G in combinations other than CpG could be disproportionately represented. To account for this possibility, CpG O:E ratios were corrected for amino acid composition across this region (Fig. 4A). A diverse distribution of CpG content was evident in viruses from every host group except ungulates, with bats in particular displaying a notable range from total suppression to overrepresentation. Otherwise, most viruses from most species still maintained some level of CpG suppression in E ORF. The exceptions with high CpG O:E ratios in E ORF were avian coronaviruses and notably, SARS-CoV and SARS-CoV-2. In contrast, other HCoVs (HCoV-229E, HCoV-HKU1, HCoV-NL63 and HCoV-OC43) all strongly under-represented CpG in E ORF, while MERS-CoV E ORF had an intermediate CpG O:E ratio of 0.6. To confirm E ORF over-represented CpG relative to the rest of the genome in SARS-CoV and SARS-CoV-2, ratios for E ORF: genomic CpG O:E were calculated (Fig. 4B). In non-bat non-avian host genomes, E ORF usually displayed CpG suppression in line with or stronger than that seen at the genome level, whereas SARS-CoV and SARS-CoV-2 starkly contrasted with this, displaying far less CpG suppression in this region. This could be linked with their recent emergence from bat reservoirs, as genome composition is more likely to be optimised for replication in that host, and the CpG composition of E ORF for both SARS-CoV-2 and SARS-CoV falls within the E ORF CpG heterogeneity apparent across bat-derived sequences.

As another check of whether differential codon usage might explain the CpG composition disparity in E ORF, we calculated CAI scores baselined against the human transcriptome. While SARS-CoV and SARS-CoV-2 E ORFs had CAI scores that were lower than those for other coronavirus E ORFs, the differences were small and did not explain the large differences in CpG ratios (Table 1).

**Table 1.** Comparison of the CpG dinucleotide composition of E ORF of coronaviruses that infect humans

Virus	CpG frequency	GC content	CpG O:E ratio	CAI
SARS-CoV-2	11	0.387	1.262	0.58
SARS-CoV	13	0.408	1.308	0.56
HCoV-229E	1	0.338	0.175	0.63
HCoV-NL63	3	0.349	0.221	0.66
HCoV-HKU1	2	0.290	0.586	0.63
HCoV-OC43	2	0.349	0.306	0.67
MERS-CoV	6	0.402	0.642	0.61

To investigate the evolutionary history of E ORF CpG composition in the HCoVs, a phylogenetic reconstruction of all 7 human coronavirus and 96 bat coronavirus E genes was performed to determine whether CpG ratios in this region were ancestrally derived. As expected (Cotten et al. 2013; Lu et al. 2020), the human viruses were interspersed among the bat viruses, reflective of their independent emergence events (Fig. 4C). The CpG compositions of the human coronavirus E ORFs, although diverse, were similar to the CpG compositions of their phylogenetically proximal bat relatives, demonstrating that CpG composition in E ORF is an ancestrally derived trait selected prior to emergence in the human population.

## 4. Discussion

We have examined the CpG O:E ratios of all the currently available complete genome sequences of coronaviruses and uncovered a noteworthy diversity. Generally, the CpG O:E ratio of coronavirus genomes from a single host species varied considerably. For bats, which serve as a coronavirus reservoir (Banerjee et al. 2019) and which had the largest number of representative sequences, the CpG O:E range was from 0.41 to 0.70, demonstrating the genome plasticity of coronaviruses and indicating that their evolution is not overtly restricted by a requirement to minimise CpG composition in the natural reservoir. The antiviral CpG-detector protein, ZAP (Takata et al. 2017), has been identified as a target for several viral proteins including the 3C protease of enterovirus 71 (Xie et al. 2018) and NS1 of influenza A virus (Tang et al. 2017)—two viruses with overall low CpG content (Atkinson et al. 2014; Gaunt et al. 2016). This highlights the importance of CpG as a pathogen-associated molecular pattern (PAMP), and so this diversity in CpG expression within the *Coronaviridae* is striking. If coronaviruses also produce a protein with anti-ZAP activity, it is possible that this has variable efficacy between strains, explaining the ability of coronaviruses to fluctuate CpG composition considerably. Alternatively (or in addition), this may be host driven; we show that average CpG suppression varies with host species (Fig. 2C) and, as previously suggested (Xia 2020), this may be linked with ZAP expression levels. We have demonstrated that CpG variation is not related to viral taxonomic grouping (Fig. 2B) but we did find an association between viral CpG composition and primary replication site, with respiratory coronaviruses having a lower CpG composition than enteric ones (Fig. 2E). This is the opposite of what has been previously suggested (Xia 2020), though this proposal was not supported by any comprehensive investigation. Nevertheless, our meta-analysis was subject to the sampling preferences of many labs who have performed surveillance for coronaviruses and many of the tissue tropism assignments we made have not been verified by experimental infections. Another limitation of this analysis is that only sequences of greater than 10 per cent divergence were included, and while this overcomes some sampling bias, we cannot assume that datapoints are independent (which is why statistical comparisons are not included). Notably, tissue tropism can be defined by much smaller divergences; for example, a deletion in the spike protein of transmissible gastroenteritis virus (a porcine coronavirus) altered the tropism of the virus from enteric to respiratory, while nucleotide identity was preserved at 96 per cent (Cox et al. 1990; Rasschaert et al. 1990). Further study on tissue tropisms of coronaviruses, as well as tissue expression profiles and antiviral activities of ZAP, is needed to validate these analyses.

Loss of CpG motifs during adaptation to the human host has been previously described for influenza A virus (Greenbaum et al. 2008), highlighting the importance of CpG composition for host adaptation. For SARS-CoV-2, we determined a genomic CpG O:E ratio of 0.408, which is similar to the human genome CpG O:E ratio of 0.2-0.4 (McClelland and Ivarie 1982; Sved and Bird 1990; Tomso and Bell 2003). Mimicry of the CpG composition of the host by ssRNA viruses is considered a mechanism to subvert detection by the innate immune response (Simmonds et al. 2013; Takata et al. 2017) and speculatively this may indicate that SARS-CoV-2 was genetically predisposed to make a host switch into humans. Similarly, the genomic CPB score of 0.048 indicates that SARS-CoV-2 uses codon pairs which are preferentially utilised in the human ORFeome, which may mean that the virus was well suited for translational efficiency in humans at its time of emergence.

In coding regions which do not have overlapping ORFs, there is no requirement at the coding level for CpG motifs to be retained (Kanaya et al. 2001). E ORF and ORF10 are not known to be in overlapping reading frames; conversely, ORF9b overlaps with the ORF for nucleocapsid (N). Some CpG retention in this region is therefore inevitable and may explain the high CpG composition of ORF9b. This nevertheless leaves open the question of why CpG motifs are retained in the E ORF and ORF10 regions (if this is not an ancestrally derived evolutionary hangover; as CpGs have not been lost from these regions between 2013 and now (Fig. 3D), this seems unlikely). CpG motifs may serve various non-exclusive purposes, including providing secondary structure (Rima and McFerran 1997), intentionally stimulating ZAP activity (by analogy with multiple viruses intentionally triggering NF-kB (Hiscott et al. 2001)), or providing m5c methylation sites (Squires et al. 2012; Khoddami and Cairns 2013; Dev et al. 2017).

It is also possible that CpG enrichment serves as a strategy for regulating translation. Conceivably, the high CpG content at the 5′ end of the E ORF transcript destines this for degradation via ZAP or CBP-associated mechanisms (Guo et al. 2007; Groenke et al. 2020) more rapidly than other viral transcripts. This could be intentional, or an evolutionarily accepted trade-off to preserve a higher importance role for CpGs. Alternatively, E ORF and ORF10 proteins may only be required late during infection (parallels with which can be drawn from the differential temporal expression and translational efficiencies of transcripts of the coronavirus mouse hepatitis virus strain A59 (Irigoyen et al. 2016)), by which time an as-yet unidentified inhibitor of ZAP (or other CpG/CBP sensor(s)) may render CpG suppression unnecessary, as suggested for human cytomegalovirus (Lin et al. 2020).

ORF9b and ORF10 do not have their own TRSs and so whether or how these ORFs are accessed is currently controversial; nevertheless, peptides from both have been identified by mass spectrometry from SARS-CoV-2-infected cells (Davidson et al. 2020). The ORF9b AUG transcription initiation site, which has a strong Kozak context (Kozak 1986), is the first AUG after and 10 nucleotides downstream of the initiation site for N ORF (which displays moderate Kozak context). It is therefore credible to think that ORF9b is accessed via leaky ribosomal scanning—a well-characterised method for accessing alternative ORFs used by coronaviruses and other viruses (Lin and Lo 1992; Chenik et al. 1995; Schneider et al. 1997; Senanayake and Brian 1997; Firth and Atkins 2010; Irigoyen et al. 2016; O'Connor and Brian, 2000; Ryabova et al. 2006; Wise et al. 2011). There is a lack of evidence that ORF10 is accessed via production of its own subgenomic RNA (Kim et al. 2020); possibly, this ORF is accessed via leaky scanning from the leader immediately preceding the N ORF. However, visual inspection of the SARS-CoV-2 genome indicated that the AUG encoding ORF10 is 24 AUGs downstream from the one initiating N ORF, making this hypothesis speculative at best. Whether the anomalous CpG composition of ORF10 is somehow involved in priming its transcription remains to be determined.

The transcript encoding E ORF incorporates an additional ∼3.4 kb of RNA and ORF10, if accessed from the transcript produced from the TRS upstream of N ORF, is present on a transcript of approximately 1.6 kb in length. Whether the described CpG enriched regions are relevant as PAMPs in these contexts is currently unclear from what is known about ZAP recognition of CpG motifs. It is also worth noting that the body TRS sequence ahead of the E gene is relatively weak in SARS-CoV-2, as it is in SARS-CoV (Marra et al. 2003), suggesting that this subgenomic mRNA may be of relatively low abundance. Of the SARS-CoV-2 transcripts which use a canonical TRS for synthesis, the donor site upstream of E ranked seventh when comparing sequencing read frequency across this site (behind reads spanning the TRS sites upstream of N, spike, ORF7a, ORF7b, ORF3a, ORF8 and M ORF respectively) in Vero cells infected at a low MOI for 24 h, indicating that E ORF is of lower abundance than most other transcripts (Kim et al. 2020). It is therefore possible that E ORF is of sufficiently low abundance for a high CpG frequency to be physiologically inconsequential. Similar logic can be applied to ORF10, which is just 117 nucleotides in length.

Synonymous addition of CpGs into a virus genome has been suggested as a potential novel approach to vaccine development by us and others (Burns et al. 2009; Atkinson et al. 2014; Gaunt et al. 2016; Moratorio et al. 2017). Here, we explore the evolutionary space occupied by coronaviruses in the context of their CpG composition and find that SARS-CoV-2 has a low CpG composition in comparison with other coronaviruses, but with CpG ‘hotspots’ in genomically disparate regions. This highlights the potential for large-scale recoding of the SARS-CoV-2 genome by introduction of CpGs into multiple regions of the virus genome as a mechanism for generation of an attenuated live vaccine. Introduction of CpG into multiple sites could also be used to subvert the potential of the virus to revert to virulence through recombination. A challenge of live attenuated vaccine manufacture is to enable sufficient production of a vaccine virus that has a replication defect. Introduction of CpGs into specific regions of the virus genome under normal circumstances can be expected to cause a viral replication defect. However, if genome regions such as conserved secondary structures and overlapping reading frames are preserved, the detrimental effects of CpG addition may be circumvented by growing virus in a ZAP-knockout system (Ficarelli et al. 2019; Odon et al. 2019), thus allowing the generation of high titre replication-defective vaccine virus stocks. 

## Supplementary data

Supplementary data are available at *Virus Evolution* online.

## Supplementary Material

veaa057_Supplementary_Data
